# Characterization and phylogenetic analysis of the complete chloroplast genome of *Actinidia latifolia* (Actinidiaceae)

**DOI:** 10.1080/23802359.2021.1881838

**Published:** 2021-03-01

**Authors:** Aihong Yang, Miao Hu, Tengyun Liu, Shujuan Liu, Hua Zhou, Faxin Yu

**Affiliations:** The Key Laboratory of Horticultural Plant Genetic and Improvement of Jiangxi Province, Institute of Biological Resources, Jiangxi Academy of Sciences, Nanchang, China

**Keywords:** *Actinidia latifolia*, complete chloroplast genome, phylogenetic analysis

## Abstract

The complete chloroplast (cp) genome of *Actinidia latifolia* was sequenced and assembled using Illumina pair-end sequencing data. The cp genome is 157,021 bp in length and comprises a large single copy (LSC) region of 88,557 bp and a small single copy (SSC) region of 21,562 bp separated by two inverted repeat (IR) regions of 23,451 bp. A total of 113 unique genes were identified, including 79 protein-coding genes, 30 tRNA genes, and four rRNA genes. Phylogenetic analysis based on cp genomes of 20 Actinidiaceae species revealed that *A. latifolia* was evolutionarily close to *A. eriantha*, *A. styracifolia*, and *A. fulvicoma.*

Kiwifruit (*Actinidia* Lindl.), a fruit genus with important economic and nutritional value, contains more than 50 species. As the frequent interspecific hybridization, species within *Actinidia* are highly variable in morphological characteristics, and their interspecific relationships are still confusing (Huang et al. 2013). *Actinidia latifolia* (Gardner & Champ.) Merr. var. *latifolia* is a representative *Actinidia* species that is widely distributed in relatively low latitude regions. It is characterized by the most flowers and fruits in an inflorescence of *Actinidia*, and the remarkably high vitamin C content in fresh fruit (Huang et al. 2013). Knowledge of the complete cp genome and phylogenetic analysis will contribute greatly to reveal the phylogeny status of *A. latifolia* and provide new insights into the evolutionary relationship and the taxonomy of *Actinidia*.

Plant material of *A. latifolia* was collected from Xunwu county (24°57′35″N, 115°34′51″E) of Jiangxi province, China. A specimen was deposited in the Herbarium of Lushan Botanical Garden, Chinese Academy of Sciences, under the voucher number LBG00148295. Total genomic DNA was extracted from fresh leaves using a modified CTAB method (Doyle and Doyle 1987) and then sequenced using the Illumina Hiseq 2000 platform. In total, 7.45 G raw reads were obtained, quality-trimmed and assembled against the plastome of *A. rufa* (GenBank: NC_039973.1) using the program NOVOPlasty (Dierckxsens et al. 2016). The annotated cp genome was submitted to GenBank with assigned accession number of MW057417.

The complete chloroplast genome of *A. latifolia* is 157,021 bp in length. It consists of two inverted repeat (IR) regions of 23,451 bp separated by the large single-copy (LSC) region of 88,557 bp and small single-copy (SSC) region of 21,562 bp. The overall GC content of the genome is 37.18%, and the GC content of the LSC, SSC, and IRa/b regions is 35.45%, 31.29%, and 43.14%, respectively. The cp genome possesses 113 unique functional genes, including 79 protein-coding genes, 30 distinct tRNA genes, and four rRNA genes. Five protein-coding genes, eight tRNA genes, and all four rRNA genes were totally duplicated in the IR regions. Of the 113 unique genes, six tRNA genes and seven protein-coding genes contain a single intron, and one gene (*ycf*3) has two introns.

We reconstructed a phylogenetic tree based on 20 complete cp genome sequences of Actinidiaceae. The maximum likelihood (ML) tree was constructed using RAxML8.0 (Stamatakis 2014) with 100 bootstrap replicates under the GTR + G model. The tree topography was generally congruent with previous reports based on genome-wide SNPs (Liu et al. 2017) and chloroplast *matK* gene (Li et al. 2002). The evolutionary analysis showed that all species with smooth-skinned fruit, such as *A. kolomikta*, *A. valvata*, and *A. macrosperma*, were located at the relatively basal positions of *Actinidia*, and *A. latifolia* was evolutionarily close to *A. eriantha, A. styracifolia,* and *A. fulvicoma* (Figure 1). The complete plastome can be subsequently used for genetic diversity studies of *A. latifolia* and will be benefit for future studies on phylogeny and taxonomy of *Actinidia*.

**Figure 1.**
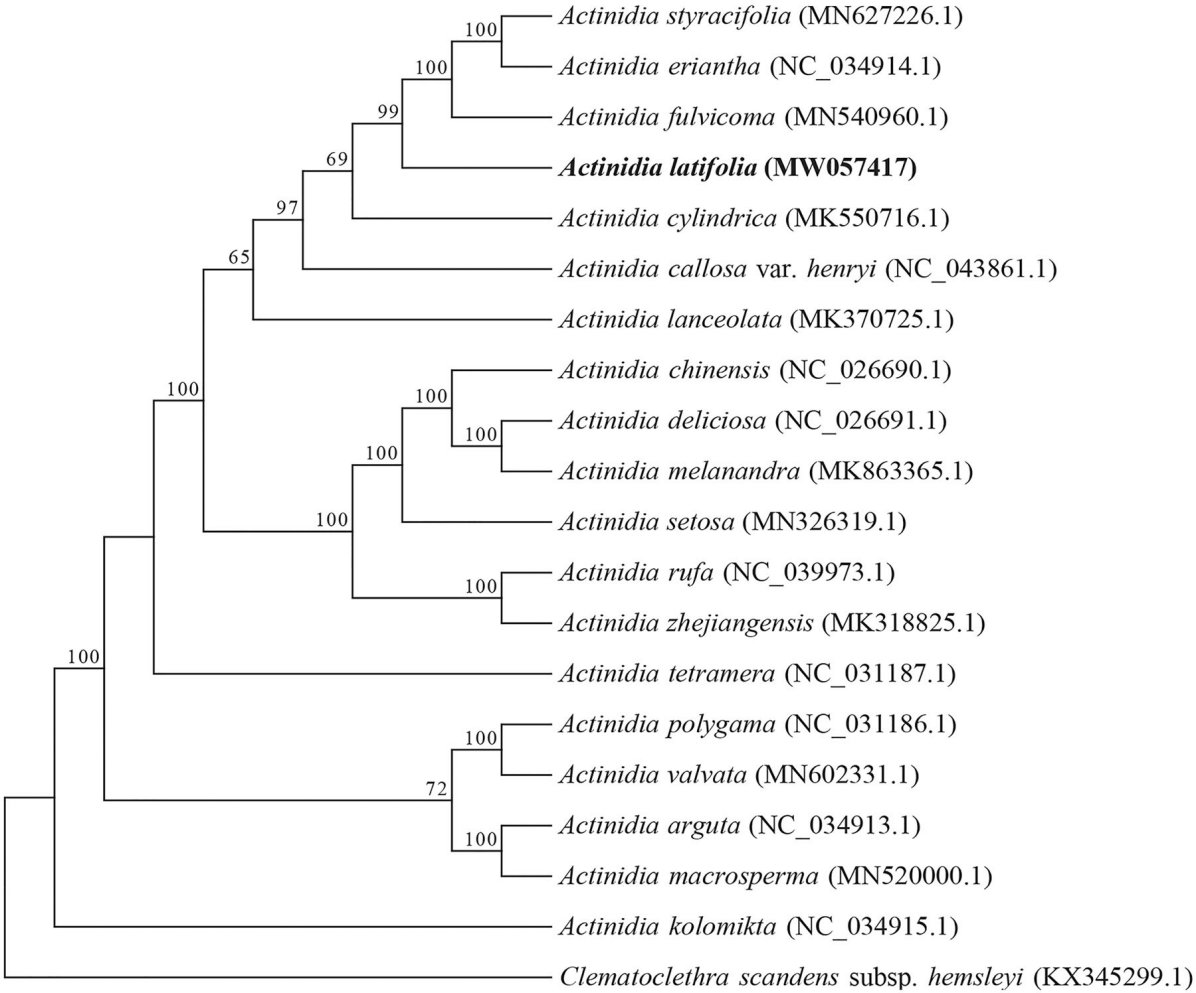
Maximum-likelihood phylogenetic tree of 20 Actinidiaceae species based on complete chloroplast genomes. Bootstrap support values above 50% are given at the nodes. *Clematoclethra scandens* subsp. *hemsleyi* was treated as an out-group.

## Data Availability

The data that support the findings of this study are openly available in GenBank of NCBI at (https://www.ncbi.nlm.nih.gov/) under the accession no. MW057417.
